# Research Progress on Mechanisms of Immune Tolerance Induced by Hepatitis B Surface Antigen in Chronic HBV Infection

**DOI:** 10.1155/jimr/6923534

**Published:** 2026-09-25

**Authors:** Li Wang

**Affiliations:** ^1^ Qishan Hospital of Yantai, 62 Huanshan Road, Zhifu District, Yantai 264001, Shandong, China

## Abstract

The sustained elevation of hepatitis B surface antigen (HBsAg) facilitates the progression of chronic hepatitis B virus (HBV) pathogenesis, and initiates and sustains host immune tolerance. Immune tolerance is a major factor contributing to viral persistence and viral clearance failure. HBsAg exerts regulatory effects on innate defenses and adaptive responses, limiting the activity of dendritic cells (DCs), natural killer (NK) cells, monocytes/macrophages, T lymphocytes, and B cells. Furthermore, it fosters a tolerogenic environment via interconnected regulatory cell networks, immune checkpoint molecules, and anti‐inflammatory cytokines. HBsAg can enhance the intrahepatic immune–tolerant (IT) microenvironment by downregulating costimulatory molecules and promoting the secretion of immunosuppressive cytokines by intrahepatic nonparenchymal cells. This review systematically summarizes how HBsAg interferes with immune cell function and signaling pathways to establish a tolerogenic environment. We also cover recent progress in treatments that target HBsAg‐induced immune tolerance. This review establishes a solid theoretical foundation and identifies new therapeutic approaches that can break tolerance to achieve a functional cure for chronic hepatitis B (CHB).

## 1. Introduction

Chronic hepatitis B virus (HBV) infection remains a significant global public health burden. It affects hundreds of millions of people around the world, and it is the main cause of liver cirrhosis, hepatic decompensation, and hepatocellular carcinoma (HCC) [1, 2]. Chronic infection develops due to the virus inducing a state of immune tolerance in the host. Hepatitis B surface antigen (HBsAg) exists in large quantities and takes part in this whole process. HBsAg is more than just a serological marker; it facilitates viral persistence and drives disease chronification [3]. The immune‐tolerant (IT) phase usually has high levels of HBsAg and HBV DNA in circulation. This phase is also marked by stable alanine aminotransferase (ALT) levels, and an absence of overt liver inflammation [4, 5]. However, new research shows subclinical immune dysregulation, and mild liver damage may occur during this phase [5].

The immunological landscape of chronic HBV infection is shaped by the interplay between viral factors and host immune responses. The innate immune response, comprising dendritic cells (DCs), natural killer (NK) cells, and monocytes, works to recognize and control HBV infection in the early stage, whereas these cells often become dysfunctional as the infection progresses to a chronic state [6, 7]. Adaptive immunity mainly comprises HBV‐specific CD4+ and CD8+ T cells responsible for clearing the virus, but they often become exhausted or functionally impaired during chronic HBV infection, leading to viral persistence [8, 9]. Persistently high levels of antigen, especially HBsAg, drive T cells toward exhaustion and trigger immune tolerance, which compromises the host’s capacity to mount an effective antiviral response [10]. Regulatory T cells (Tregs) and myeloid‐derived suppressor cells (MDSCs) expand in number, fostering an immunosuppressive microenvironment in chronic HBV infection [11, 12].

HBsAg is produced by covalently‐closed circular DNA (cccDNA) in infected hepatocytes and also by cells that harbor integrated HBV DNA (iDNA) [13]. When HBsAg persists staying in the body, it causes virus‐specific T cells to stop functioning normally, damages the functions of DCs and monocytes, and helps regulatory immune cells multiply. This eventually creates an immunosuppressive environment that warrants viral persistence [3, 14]. A previous study has demonstrated that in HCC patients, higher serum HBsAg levels correlate negatively with the clinical efficacy of immune checkpoint inhibitor (ICI) therapies [15]. HBV S gene mutation can change the structure of HBsAg, and such alterations may affect how the immune system recognizes the virus [16]. These findings underscore the imperative for therapeutic interventions designed to break the immune tolerance induced by HBsAg.

Advances in immunology and biotechnology have enabled the development of new strategies that target HBsAg‐mediated immune tolerance. These methods cover therapeutic vaccines built to trigger strong HBV–specific T‐cell and B‐cell immunity, checkpoint blockade treatments that target programmed cell death protein 1 (PD‐1)/PD‐L1 and TIGIT axes, and small molecules that boost innate sensing through the STING pathway [17–19]. Fc‐optimized monoclonal antibodies targeting HBsAg have shown that they can accelerate antigen clearance, and they also increase T‐cell reactivity [20]. Present investigations evaluate synergistic regimens that couple antigen‐suppressing agents—such as small interfering RNA (siRNA) or nucleic acid polymers (NAPs)—with immunomodulatory strategies; in concert, these interventions break immune tolerance and pave the way toward a functional cure [21, 22]. Other approaches work to modulate the composition of regulatory cells and the cytokine environment. This is achieved by depleting or inhibiting Tregs and MDSCs, and by utilizing adjuvants that promote DC activation and sustain T follicular helper (Tfh) cell function. This in turn brings effective protection against the virus [11, 12, 23].

Continued research is needed because patients have different immune responses, viral antigen load interacts with immune exhaustion in varied ways, and precise biomarkers are required to guide therapy [8, 24].

## 2. Biological Characteristics of HBsAg and Its Role in Immune Tolerance

### 2.1. Molecular Structure and Secretion Forms of HBsAg

Structurally, HBsAg is made up of three envelope proteins, the small S protein, the middle M protein, and the large L protein, which are encoded by the S gene. These proteins assemble together to form subviral particles (SVPs). The quantity of produced SVPs far exceeds that of infectious Dane particles, which are complete HBV virions. SVPs work as a decoy antigen reservoir, depleting neutralizing antibodies and specific B cells [25, 26].

Genotypic variations and mutations in the S gene have a great impact on the efficiency of HBsAg secretion and its antigenicity [27]. Impaired HBsAg secretion can lead to intracellular accumulation of the protein, resulting in occult HBV infection (OBI), defined by the presence of viral DNA in the absence of detectable serum HBsAg via conventional assays [28, 29]. Specific mutations such as C138R and N320 can affect secretion, antigenicity, and immunogenicity, influencing diagnostic detection and immune recognition simultaneously [30, 31]. HBsAg secretion needs complicated pathways to finish intracellular trafficking. Changes to HBsAg production and release are connected to endoplasmic reticulum (ER) stress and autophagy. Autophagic flux facilitates the release of SVPs and naked capsids [32]. When substances such as thapsigargin or stearic acid trigger ER stress, HBsAg and HBV DNA build up inside cells because secretion is blocked. The process also helps the virus replicate [33, 34]. When HBsAg binds to antibodies such as hepatitis B immunoglobulin (HBIG), antigen‐antibody complexes precipitate in multivesicular bodies, reducing the amount of HBsAg [35]. The HBsAg derived from iDNA exhibits lower secretion efficiency than that derived from cccDNA [36]. With increased duration of NA treatment and decreased cccDNA‐containing cells, producing HBsAg mRNA transcription shifted from chiefly cccDNA to chiefly iDNA [13].

Therapeutically, researchers have identified small molecules and natural compounds that can decrease HBsAg levels by interfering with its secretion and assembly [37, 38]. Engineered chimeric antigen receptors, that target HBV envelope proteins, can inhibit HBsAg secretion and viral budding [39].

### 2.2. HBsAg as a “Tolerogen”: Immunological Characteristics

The persistently elevated levels of HBsAg may lead the immune system to recognize it as a “self” or harmless antigen, establishing a condition of immune tolerance, rather than an effective antiviral response. This phenomenon is particularly evident during the immune tolerant (IT) phase of chronic HBV infection, characterized by a minimal inflammatory response, that may paradoxically delay the development of HCC [40, 41]. Such persistent antigen stimulation triggers different mechanisms of immune dysfunction, including clonal deletion, anergy, and exhaustion of HBV‐specific T cells and B cells. For example, research conducted on HBsAg‐transgenic mouse models shows that CD8+ T cells specific for HBsAg drop sharply or lose normal function. Tregs that carry inhibitory receptors such as PD‐1 and TIGIT further suppress effective antiviral responses [42]. On the molecular level, HBsAg interacts with antigen‐presenting cells (APCs), including DCs and macrophages, thereby affecting cell maturation and antigen‐presenting capacity. The tolerogenic environment is characterized by insufficient activation of these APCs, which results in insufficient stimulation of HBV‐specific T helper (Th) and cytotoxic T lymphocyte (CTL) responses. This tolerogenic state is exacerbated by the formation of HBsAg immune complexes (HBsAg‐CICs), which can alter the antigen processing and further impair immune recognition [16].

In clinical settings, elevated serum levels of HBsAg correlated with impaired antiviral immune responses. In contrast, a reduction in HBsAg levels is associated with immune activation and potent antiviral responses. This phenomenon has been observed in patients receiving antiviral therapy as well as those undergoing immune checkpoint blockade [43, 44]. High HBsAg levels lead to the upregulation of inhibitory receptors, including PD‐1 on T cells and FcRL5 on B cells. This exhausted profile can be partially reversed through PD‐1/PD‐L1 blockade [43, 44].

Recent advances in strategies have shown that engineered fusion proteins combining anti‐PD‐L1 antibodies with interferon‐alpha (IFN‐α) can increase antigen uptake, promote the maturation of macrophages and DCs, and trigger strong HBV–specific T‐ and B‐cell responses in tolerant mouse models [44, 45]. In therapeutic vaccination, adjuvants targeting DCs and novel delivery systems, such as liposomes and microneedles, are being investigated to enhance antigen presentation and induce effective immunity [46, 47].

## 3. Inhibitory Impact of HBsAg on the Innate Immune System

The innate immune response plays a crucial role in establishing an adequate immune response against the virus at the initial phase of HBV infection. However, HBV employs various mechanisms to evade the host’s innate immunity, which partly contributes to the chronicity of infection [48]. Importantly, HBsAg regulates innate immune cell function via multiple mechanisms, including inhibiting NK cell activity, interfering with macrophage polarization, and impairing DC maturation, thereby disrupting antiviral immune responses. At the molecular level, HBsAg interferes with pattern recognition receptor (PRR) signaling pathways, such as Toll‐like receptor (TLR) and retinoic acid–inducible gene I (RIG‐I) pathways, regulating the balance between proinflammatory and anti‐inflammatory cytokines (e.g., inhibiting IFN‐α/β and interleukin‐12 (IL‐12) secretion while promoting IL‐10 and TGF‐β production) and establishing an immunosuppressive cytokine network, thereby suppressing the interferon system [49–51].

### 3.1. Regulation of DC Function

DCs are pivotal APCs that orchestrate immune responses, particularly in antiviral immunity. They are broadly classified into three main subsets: classical or conventional DCs (cDCs), plasmacytoid DCs (pDCs), and monocyte‐derived DCs (Mo‐DCs), which contribute differentially to immune defense against HBV infection [50, 52]. A previous study demonstrated that pDCs and mDCs exhibited impaired maturation in chronic hepatitis B (CHB) patients with higher baseline HBsAg levels than in patients with lower baseline HBsAg levels, regardless of changes in these levels following treatment [50]. In HBV‐transgenic (HBV‐Tg) mice, intrauterine exposure to HBsAg compromised DC generation and activation, leading to a subsequent suboptimal vaccine response [53]. Circulating and hepatic pDCs and cDCs exhibited impaired maturation following stimulation with specific TLR agonists in CHB patients, and markedly reduced capacity of circulating DC subsets to produce IL‐12p70, tumor necrosis factor‐α (TNF‐α), IFNα, IFNλ1, and IFNλ2. Furthermore, most of these dysfunctions were correlated with HBsAg and HBV DNA levels [54].

C‐type lectin receptors (CLRs) are key receptors used by DCs to orchestrate responses to pathogens. The CLR repertoire of circulating and intrahepatic cDC and pDCs was perturbed, with some candidate CLRs identified as responsible for HBsAg binding to cDCs (CD367/DCIR/CLEC4A and CD32/FcɣRIIA) and pDCs (CD369/DECTIN1/CLEC7A and CD336/NKp44). Furthermore, HBsAg impaired DC functions in a CLR‐ and glycosylation‐dependent manner [55]. Although some studies demonstrated that HBsAg exposure favored DC activation, it significanly modulated TNF‐related apoptosis–inducing ligand (TRAIL) expression in response to TLR ligands and increased the secretion of cytokines/chemokines involved in immune tolerance. HBsAg further suppressed metabolic activity of DC subsets while inducing metabolic reprogramming [56]. The metabolic shift leads to increased release of immunoregulatory cytokines, including TGF‐β, and chemokines such as CX3CL1, promoting the establishment of an immunosuppressive environment within the liver [56]. However, another study found that the recognition of HBsAg by DCs involves soluble CD14 (sCD14) and TLR4. Exposure of peripheral blood–derived BDCA1(+) mDC to HBsAg induces robust DC maturation, cytokine production, and enhanced capacity to activate virus‐specific CTLs via an sCD14‐dependent mechanism. The abundance of sCD14‐HBsAg complexes varies across CHB stages, which have important implications for immunity to HBV infection in neonates given the naturally low sCD14 levels [57]. pDCs from CHB patients had lower OX40L expression as well as impaired capacity for Tfh induction compared with those from healthy donors [58]. The interaction between DC and NK cells is also highly relevant in fighting against HBV. TLR9 ligand (TLR9‐L)–activated pDCs from CHB patients cannot induce cytolytic activity of NK cells. This altered function of pDCs was also associated with reduced expression of OX40L [59].

Emerging therapeutic strategies have demonstrated that engineered fusion proteins targeting immune checkpoints, such as PD‐L1, synergize with IFN‐α to stimulate DCs and increase their capacity for antigen uptake and maturation, improving presentation of HBsAg to T cells and fostering robust HBV–specific T‐cell immunity [44, 45]. Adjuvanted therapeutic vaccines combining HBsAg with potent immunostimulants, such as CpG oligodeoxynucleotides and QS‐21, have been shown to amplify DC activation and maturation, upregulate costimulatory molecule expression, and induce cytokine release that favor Th1 polarization, which can effectively restore the capacity of DCs to prime vigorous HBV–specific CD4+ and CD8+ T‐cell responses. This revitalized immune activity reduces circulating HBsAg levels and reverses T‐cell exhaustion [60]. TLR7 agonist treatment can restore pDC function, thereby enhancing the secretion of IL‐6 and IL‐12 and promoting the differentiation of IL‐21–producing Tfh cells [58].

Collectively, therapeutic approaches designed to restore DC functionality encompass immune checkpoint blockade, adjuvant‐enhanced vaccination, TLR agonism, and metabolic reprogramming. These strategies hold significant promise to overcome this tolerogenic state and elicit robust antiviral immunity. Elucidating the precise molecular and cellular pathways through which HBsAg modulates DC activity is critical for the rational design of these interventions and the optimization of clinical outcomes in patients with CHB [58–62].

### 3.2. HBsAg Suppression of NK Cell Function

Activated NK cells with intact cytotoxicity and cytokine production capacity are essential for the clearance of HBV‐infected hepatocytes and for the activation of adaptive immune responses, including DC maturation and T‐cell priming. Studies have shown that NK cells in CHB patients with low HBsAg levels (<100 IU/mL) display an activated phenotype with increased expression of the activation markers CD38 and granzyme B, as well as the proliferation marker Ki‐67. In patients with high HBsAg titers, NK cells display reduced degranulation, diminished IFN‐γ secretion, and an exhausted phenotype [63]. In patients with high HBsAg levels, the expression of the activating receptor NKG2D on NK cells is significantly downregulated, while the inhibitory receptor CD94/NKG2A is upregulated, accompanied with a reduced TNF‐α and IFN‐γ cytokine levels and impaired cytotoxic capacity to eliminate HBV‐infected hepatocytes [49, 64]. HBsAg can inhibit STING expression, thereby disrupting NK cell function via the STAT3‐STING axis, and suppress the NK cell response to cGAMP [65].

HBsAg could suppress mTOR signaling activity triggered by IL‐15 through competitively binding to the IL‐15 receptor β (IL‐15Rβ, CD122) on NK cells, resulting in downregulation of HIF‐1α and its downstream genes. Intravenous injection of an HBsAg‐neutralizing antibody or intraperitoneal injection of the mTOR agonist MHY1485 restored IL‐15/mTOR signaling and NK cell activation in HBV‐carrier mice [66]. NK cells from vaccinated subjects exhibited enhanced cytotoxic and proliferative responses against autologous HBsAg–pulsed moDCs compared with those from unvaccinated subjects. This cytolytic activity was significantly more potent against HBsAg‐pulsed moDCs than against HBcAg‐pulsed moDCs in a NKG2D‐dependent manner [67].

### 3.3. Monocytes/Macrophage

In chronically HBV–infected patients, monocytes exhibit multitude defects including alteration in TLR expression, phagocytic activity, cytokine production, migratory ability, polarization of monocyte‐derived macrophages, and monocyte‐T‐cell interaction. High levels of HBsAg and IL‐4 can potentiate these defects via β‐catenin induction [7]. HBsAg can upregulate immune checkpoint molecules on the surface of monocytes, such as Galectin‐9 (Gal‐9) and PD‐L1. Gal‐9+ monocytes can promote the expansion of CD4+CD25+FOXP3+ Tregs and CD19+IL‐10+ regulatory B cells (Bregs) and preferentially differentiate into M2‐type macrophages, which further suppress the antiviral immune response [14]. Pretreatment monocyte cell line THP‐1 with HBsAg leads to a significant upregulation of A20 (a ubiquitin‐editing enzyme), inhibiting LPS‐induced activation of nuclear factor‐kappa B (NF‐κB) and mitogen‐activated protein kinase (MAPK), thereby blocking the TLR4 downstream signaling pathway, leading to reduced production of proinflammatory cytokines (such as TNF‐α and IL‐6) [68]. HBV can employ HBsAg to induce suppressive monocytes expressing HLA‐E, PD‐L1, IL‐10, and TGF‐β via the myeloid differentiation factor 88 (MyD88)/NF‐κB signaling pathway. Such suppressive monocytes can initiate regulatory NK cell differentiation via PD‐L1 and HLA‐E signaling to produce IL‐10, resulting in T‐cell inhibition [69].

### 3.4. Interference With Innate Immune Signaling Pathways

HBsAg induces immune tolerance by interfering with innate immune signaling pathways, a critical mechanism underlying the persistence of chronic HBV infection and disease progression. HBsAg can interfere with essential PRR‐mediated signaling cascades both in hepatocytes and in immune compartments. Key PRRs here include TLRs, RIG‐I–like receptors, NOD‐like receptors (NLRs), and CLRs, as well as the cGAS‐STING pathway, which play a pivotal role in detecting viral components and initiate antiviral defenses, primarily through the induction of type I IFNs and proinflammatory cytokines [55, 65, 68, 70]. Accumulating evidence indicates that HBsAg inhibits type I IFNs and inflammatory mediator production by disrupting TLR and RIG‐I pathways through multiple mechanisms. HBsAg blocks downstream adaptor molecules including MyD88 and mitochondrial antiviral signaling protein (MAVS), which are required to activate transcription factors such as interferon regulatory factor 3 (IRF3), IRF7, and NF‐κB [67]. A recent study demonstrated that HBsAg disrupted NF‐κB promoter activity via ectopic expression of MyD88 and TAK1. Furthermore, HBsAg inhibited both the LPS‐induced polyubiquitination of tumor necrosis factor receptor–associated factor 6 (TRAF6) and the formation of the TRAF6‐TAB2 complex by regulating A20. Downregulation of A20 via siRNA restored LPS‐mediated cytokine production, suggesting its crucial role in HBsAg‐mediated inhibition of TLR4 signaling pathway [68]. HBsAg can specifically bind to the TAK1‐TAB2 complex, inhibiting its phosphorylation and ubiquitination modifications, thereby blocking the activation of the NF‐κB pathway. The expression of TAK1 and TAB2 and the translocation of NF‐κB inversely correlated with HBsAg levels in clinical liver tissues [51]. HBsAg interacts with the kinase domain of TANK‐binding kinase 1 (TBK1), thereby disrupting the TBK1‐IRF3 complex and inhibiting type I interferon production. In addition, this process crosstalks with autophagy and metabolism [70]. These transcriptional regulators control the expression of type I IFNs and inflammatory cytokines at the genetic level [51, 70–72].

Studies have demonstrated that α2,6‐biantennary sialoglycans of HBsAg facilitate high‐affinity binding to inhibitory receptors such as myeloid ligand of C‐type lectin (mitomitogen) III (SIGLEC‐3, CD33), which leads to phosphorylation of tyrosine residues within immunoreceptor tyrosine–based inhibitory motifs (ITIMs) and promotes the recruitment of SHP‐1 and SHP‐2 to enhance immunosuppressive signals. Targeting SIGLEC‐3 with an anti‐SIGLEC‐3 mAb abolishes such inhibition and thus restores antigen presentation and cytokine production capabilities in response to the TLR‐7 agonist GS‐9620 in PBMCs [73, 74].

Despite significant progress in the study of individual pathways, the cross‐regulatory networks among these pathways have not yet been fully elucidated. Furthermore, the development of inhibitors targeting HBsAg and the utilization of PRR agonists to restore innate immune function remain both a critical research focus and a substantial challenge in current research.

## 4. Suppression of Adaptive Immune T‐Cell Responses by HBsAg

### 4.1. CD8+ T‐Cell Exhaustion and Dysfunction

Sustained HBV antigenic stimulation induces the upregulation of multiple inhibitory receptors on HBV‐specific CD8+ T cells, which including PD‐1, T‐cell immunoglobulin and mucin domain–containing protein 3 (TIM‐3), and CTL‐associated antigen 4 (CTLA‐4). This hypofunctional state is characterized by impaired proliferative capacity, diminished production of key effector cytokines such as IFN‐γ and TNF‐α, and reduced cytolytic activity against target cells [75–77]. This state of exhaustion is not a transient loss of function. It drives lasting epigenetic alterations and transcriptional rewiring that turn CD8+ T cells into a refractory status, rendering the cells unresponsive to conventional stimulatory cues such as IL‐2 [78, 79]. Recent studies have elucidated the substantial heterogeneity in exhausted CD8+ T‐cell pools during chronic HBV infection. Subsets expressing the chemokine receptor CCR5 exhibit only partial exhaustion and retain superior antiviral potency compared to CCR5‐negative cells [80]. The transcription factor TOX orchestrates this dysfunctional transcriptional program. TOX expression levels are positively correlated with viral load and the severity of CD8+ T‐cell impairment. TOX expression persists even after effective viral suppression, leaving a lasting molecular mark from chronic infection [81]. Other transcriptional modulators such as Eomesodermin (Eomes) upregulate the surface expression of inhibitory checkpoints, including PD‐1, LAG‐3, and CD160, thereby further maintaining the exhausted phenotype and decreasing cytokine secretion [77]. In contrast, some studies have shown that HBV‐specific T‐cell responses and HBsAg‐specific T‐cell numbers were not affected by HBsAg levels but rather by age [82, 83].

In the context of chronic HBV infection, mitochondrial reactive oxygen species (ROS) accumulate within CD8+ T cells, and this is closely associated with elevated PD‐1 levels and reduced IFN‐γ output. This association suggests that impaired mitochondrial function drives T‐cell exhaustion [84]. As a key glycolytic enzyme, enolase governs the progression of exhaustion by regulating cell metabolism. In the absence of enolase, glycolysis is impaired, and T cells can no longer function optimally. Restoring these inhibited metabolic steps allows T cells to regain their antiviral their antiviral capacity [85]. Pharmacological inhibition of specific metabolic pathways, such as that of acyl‐CoA:cholesterol acyltransferase (ACAT), facilitates the functional restoration of exhausted HBV‐specific CD8+ T cells. ACAT inhibition strengthens T‐cell receptor (TCR) signaling and enhances cellular energy metabolism, thereby potentiating the host antiviral immune response [78].

Beyond intrinsic metabolic defects, the hepatic microenvironment actively promotes CD8+ T‐cell exhaustion through intertwined immunoregulatory networks during chronic HBV infection. A recent study demonstrated that the degree of impaired intrahepatic T‐cell function did not correlate with the HBsAg levels in the premalignant stage [77]. Mesencephalic astrocyte–derived neurotrophic factor (MANF) drives the expansion of MDSCs via the IL‐6/STAT3 axis and indirectly promotes the CD8+ T‐cell dysfunction [86]. Concurrently, NK cells modulate CD8+ T‐cell function through the Gal‐9/TIM‐3 interaction. Surface Gal‐9 on NK cells binds to TIM‐3 on CD8+ T cells, thereby reducing cytokine production and promoting apoptosis [87]. Heightened cAMP‐PKA signaling in hepatic CD8+ T cells, induced by crosstalk with liver sinusoidal endothelial cells (LSECs), impairs TCR transduction and effector functions. This mechanism constitutes a liver‐specific immune regulation system that attenuates antiviral responses in an orderly manner [88].

Therapeutic strategies that reverse CD8+ T‐cell exhaustion have focused on immune checkpoint blockade, therapeutic vaccination, and antigen‐load reduction strategies. Inhibiting the PD‐1/PD‐L1 pathway partially restores HBV‐specific CD8+ T‐cell function, particularly for patients with lower viral loads and lack hepatitis B e antigen (HBeAg). Clinical outcomes remain highly variable, strongly influenced by the specific stage of exhaustion and phase of disease [89, 90]. Therapeutic vaccines utilizing HBsAg with adjuvants such as CpG 1018S and QS‐21 can expand populations of HBV‐specific CD8+ T cells, reduce the expression of exhaustion markers such as TIM‐3 and TIGIT, and restore the production of polyfunctional cytokines, thereby effectively breaking immune tolerance [57]. The clearance of circulating HBsAg alone does not boost CD8+ T‐cell activity significantly, indicating that immune exhaustion develops through multiple mechanisms that go beyond simple antigen exposure [91]. Preclinical data indicate that combining siRNA‐mediated antigen suppression with therapeutic vaccination synergistically enhances the generation of polyfunctional HBV–specific CD8+ T cells and promotes viral clearance. This confirms that lowering the antigen burden contributes to reversing exhaustion [92]. In the context of coinfection, particularly especially HBV/HIV coinfection, CD8+ T cells exhibit a stem‐like precursor exhausted phenotype (Tpex) marked by the expression of TCF‐1 and PD‐1. This phenotype maintains cellular plasticity and responsiveness to checkpoint inhibitors, a characteristic that contrasts sharply with the terminally exhausted state observed in HBV mono‐infection. The retention of stemness facilitates a stronger antiviral response and provides useful direction for developing personalized immunotherapies [93].

Although the removal of antigen stimulation alone is not sufficient to restore the function of exhausted T cells, the reduction of viral antigen load facilitates the therapeutic activation of functional antiviral immunity in chronic HBV carriers [94]. Notably very low HBsAg levels influence T‐cell response to anti‐PD‐L1 treatment [83].

### 4.2. CD4+ Th Cell Dysfunction

The distinct subsets of CD4+ Th cells are necessary to orchestrate a robust immune response against viral infections. HBsAg strongly limits Th1 immune responses by reducing the production of IFN‐γ and TNF‐α, which reduces the activation of cytotoxic CD8+ T cells and macrophages, thereby impairing the clearance of HBV‐infected hepatocytes [43, 58]. Tregs express surface CD25 and transcription factor Foxp3, and their numbers increase in chronic HBV infection, accompanied by increased secretion of anti‐inflammatory cytokines, such as IL‐10 and TGF‐β [14, 95].

The establishment of this immunosuppressive milieu is further reinforced by contact‐dependent mechanisms mediated via inhibitory receptors, specifically PD‐1 and CTLA‐4, which are coexpressed on Tregs and effector T cells [14, 96]. Elevated Treg frequencies were associated with higher expression of exhaustion‐associated markers in CD4+ and CD8+ T‐cell populations, namely include PD‐1, TIM‐3, LAG‐3, and TOX [97, 98]. The tfh cells are essential for the production of high‐affinity antibodies. During chronic HBV infection, impaired Tfh cells restrict B‐cell function and cause defects in humoral immune responses, compromising the host’s ability to control virus effectively [98].

Hyperactive Tregs can suppress effector T‐cell responses and indirectly impair Tfh cell function. The impaired Tfh cell response to HBsAg in CHB patients can be restored by depletion of Treg cells or blockade with an antibody against CTLA4 [99]. In preclinical models, therapeutic vaccination protocols that potently activate CD4+ T cells during the initial immunization phase enhance HBV‐specific antibody production and CD8+ T‐cell activity, thereby leading to superior viral control [17, 60]. Checkpoint inhibitor therapies targeting the PD‐1 and CTLA‐4 pathways hold promise for restoring CD4+ T‐cell function and promoting seroconversion in patients with CHB [96, 97]. The combination of immune checkpoint blockade and therapeutic vaccines exerts a synergistical effect to expand CD4+ T‐cell–mediated immunity, effectively counteracting HBsAg‐induced tolerance. Notably, experimental data show that removing CD4+ T cells, which includes the Treg subset, can improve CD8+ T‐cell–mediated HBV suppression in an unexpected way. These findings suggest that precise modulation of specific CD4+ T‐cell populations could be an effective strategy to break immune tolerance [42, 100].

In summary, HBsAg‐driven immune tolerance induces a skewed CD4+ Th cell distribution in chronic HBV infection. Attenuated Th1 responses coincide with expanded Tregs and impaired Tfh cell function. Elucidating the molecular mechanisms underlying this CD4+ T‐cell exhaustion is critical for the design of new immunotherapies.

### 4.3. Induction of Central T‐Cell Tolerance

The establishment of T‐cell tolerance to HBsAg relies on sophisticated mechanisms that operate in the thymus and in peripheral tissues. This process is particularly critical in models of perinatal HBV transmission [101]. Central tolerance is mediated predominantly by thymic negative selection. This process eliminates developing thymocytes that express high‐affinity TCRs specific to self or persistent antigens, thereby preventing autoimmunity and maintaining immune homeostasis [102]. In chronic HBV infection, new findings indicate that HBsAg translocate to the thymic medulla, where monocytic MDSCs (mMDSCs)—which enter the thymus via CCR9‐CCL25 chemokine signaling—mediate antigen cross‐presentation. Specifically, HBsAg upregulates CCR9 expression on mMDSCs via the activation of the ERK1/2 pathway and IL‐6 signaling. This process induces clonal deletion of high‐affinity CD8+ thymocytes specific to HBsAg via negative selection, thereby eliminating the majority of HBV‐specific T‐cell precursors [101]. This effect has been observed in CHB patients and murine models. In these experiments, the adoptive transfer of mMDSCs eliminated only HBsAg‐specific CD8+ T cells via a CCR9‐dependent mechanism [101]. These results demonstrate a new pathway that allows central tolerance to HBV antigens to form during the early stage of viral contact.

## 5. Impact of HBsAg on B Cells and Humoral Immunity

Humoral immunity targeting HBsAg plays a critical role in viral clearance and achieving a clinical cure. The vast quantity of HBsAg SVPs that far exceeds the number of infectious virions saturates and activates HBsAg‐specific B cells, eventually rendering them dysfunctional. This process prevents B cells from differentiating into plasma cells capable of secreting virus‐neutralizing antibodies. Even when antibodies are produced, the resulting immunoglobulins often exhibit weak binding affinity for the virus or fail to neutralize it entirely. Consequently, they cannot clear circulating systemic virus particles, leading to viral persistence within the host [103]. B‐cell function is further impaired in the immunosuppressive environment characteristic of chronic HBV infection. B cells exhibit increased expression of inhibitory receptors on their surface. A recent study found that CTLA4 was highly upregulated in both peripheral and hepatic HBsAg–specific B cells from CHB patients. The diminished IL‐6 JAK/STAT3 and IL‐2/STAT5 signaling pathways contribute to the inability to secrete HBsAb [104] even. This poor antibody response stem from both intrinsic defects in B cells and in sufficient help from T cells. This assistance is primarily provided by CD4+ Tfh cells, which are required to enable full B cells activation and the development of higher binding affinity. CHB patients exhibited significantly higher frequencies of total Tfh cells, lower frequencies of Tfh17 cells, and elevated PD‐1 expression levels. The quiescent Tfh17 cell frequency negatively correlated with HBsAg^+^B cells. Activated Tfh17 cells correlated positively with HBsAg levels, while quiescent Tfh17 cells correlated negatively with HBsAg levels. Therefore, activated Tfh17 cells in CHB patients may lead to B‐cell differentiation into plasma cells [105]. In contrast, another study shows that distinct serum HBsAg levels only minimally modulate the gene expression profiles of B cells [106]. Research shows that vaccine strategies that restore HBV‐specific CD4+ T‐cell responses can enhance antibody levels. These findings indicate that cellular immunity and humoral immunity are interdependent to control HBV effectively [107].

Strategies that aim to reduce circulating HBsAg such as capsid assembly modulators (CAMs) and siRNA show promise in restoring B‐cell function exhausted by excessive antigen exposure. This can stimulate the production of neutralizing antibodies and help build strong adaptive immunity in polyfunctional T cells [91, 108].

Emerging evidence suggests that the gut microbiota and its interaction with Peyer’s patches influence systemic adaptive immunity against HBV, and that altering these interactions indirectly influences B‐cell dynamics. In the presence of intestinal dysbiosis, adaptive immune function is compromised, impeding HBV antigen clearance. Microbial signals aid the body in maintaining robust humoral responses. Moreover, immunomodulatory agents, such as probiotics and their metabolic derivatives, can boost CD4+ T‐cell–mediated immunity, which in turn may enhance B‐cell function and antibody production [109].

## 6. Shaping and Maintenance of the Intrahepatic Immune Tolerant Microenvironment

The liver is a highly tolerogenic organ that plays a critical role in the host’s HBsAg‐induced immune tolerance. This intrinsic tolerogenicity results from the unique cellular composition of the liver and the hepatic microenvironment. The liver harboring different nonparenchymal cell populations can serve as APCs but are poorly efficient in T‐cell activation, with a propensity to induce T‐cell tolerance. This is because they exhibit low expression of costimulatory molecules, upregulated coinhibitory ligands PD‐L1 and PD‐L2 upon IFN stimulation, and increased production of immunosuppressive cytokines, such as IL‐10 and TGF‐β [110]. These cells include resident DCs, LSECs, Kupffer cells (KCs), hepatic stellate cells (HSCs), and hepatocytes themselves. Additionally, the elevated levels of soluble mediators, such as arginase, indoleamine 2,3‐dioxygenase (IDO), and suppressive cytokines; the upregulation of inhibitory checkpoint receptor‐ligand pairs; and the expansion of regulatory cells, such as CD4+FOXp3+ Treg cells, MDSCs, and NK cells, collectively contribute to T‐cell functional exhaustion in the chronically infected liver [50, 110]. Such cell populations are strategically situated in hepatic sinusoids to continuously take up antigens and gut‐derived microbial products transported via the portal circulation into the hepatic microcirculation, maintaining a delicate equilibrium between immune activation and suppression, a balance essential for preventing deleterious inflammatory cascades and consequent liver injury [111].

Hepatic macrophages comprise two major subsets: embryo‐derived KCs and recruited macrophages derived from peripheral blood monocytes (MoMFs). KCs can be partially replaced by infiltrating MoMFs during acute or chronic liver injury [112]. Under HBsAg stimulation, KCs tend to polarize from a proinflammatory (M1) towards an anti‐inflammatory (M2) phenotype, characterized by the secretion of IL‐10 and TGF‐β, which are crucial mediators that promote immune tolerance within the liver microenvironment. Mechanistically, HBsAg upregulates Sirtuin 1 (SIRT1) deacetylase expression, promoting the deacetylation of the Notch1 intracellular domain (NICD), ultimately leading to decreased NF‐κB nuclear translocation in macrophages [113]. The expression of CD163, which marks M2‐polarized KCs, was elevated in the liver of CHB patients. In an HBV‐persistent mouse model, CD163 deficiency reduced KC‐derived IL‐10 secretion [114]. A study indicated that KCs could internalize HBsAg in vitro and induce proinflammatory cytokine production without altering their phenotype [115]. However, recent research found that HBsAg‐carried miR‐939 in SVPs enters monocytes and significantly augments IL‐8 production through the MAPK p38 signaling pathway, contributing to liver necro‐inflammation [116].

Studies have demonstrated that macrophage migration inhibitory factor (MIF) expression is significantly upregulated in HSCs following stimulation with HBsAg. MIF enhances the TGF‐β/SMAD signaling pathway through its receptor CD74, promoting HSC activation and liver fibrosis progression [117]. Upon interacting with HBsAg and other viral antigens, potent immunoinhibitory effectors, such as PD‐L2 and IDO, are also upregulated by HSCs, which are responsible for the inhibitory effect on the proliferation response of T cells and the expansion of Tregs. This synergy of HSCs with LSECs and KCs is of paramount importance for maintaining immune tolerance during chronic HBV infection [51, 118–120].

LSECs, with their distinct fenestrated structure and low expression of costimulatory molecules, actively induce the generation of Tregs and inhibit effector T‐cell function [121]. HBsAg can further downregulate the expression of MHC class II molecules and of costimulatory molecules, such as CD80 and CD86, on the surface of LSECs, thereby impairing their ability to present antigens to CD4+ T cells [54]. The functions of LSECs and other nonparenchymal cells collectively form a complex synergistic network, shaping the liver’s unique immune tolerance microenvironment, particularly under the influence of HBsAg [122]. TLR‐induced expression of IFN‐γ in murine KCs and LSECs was markedly suppressed in the presence of HBsAg, whereas the expression of IL‐10 was enhanced. The activation of NFκB, IRF‐3, and MAPKs in these liver cells was potently suppressed by HBsAg. The suppression of T‐cell activation by HBsAg, mediated via TLR3‐stimulated KCs or LSECs, could be reversed by anti‐IL‐10 antibodies [68]. A recent study shows that HBsAg can drive T‐cell immunity through noncanonical antigen presentation. The intrahepatic cDC subset cDC1 could present HBsAg by MHC‐I cross‐dressing, driving the CD8^+^T‐cell response [123].

## 7. Progress on Combination Strategies at Breaking HBsAg‐Induced Immune Tolerance

Therapeutic strategies targeting the reduction of HBsAg levels combined with other immunomodulatory methods have proven promising. HBV siRNA pretreatment prior to therapeutic vaccination and PDL1 blockade in combination with cytokines IL‐2 or IL‐15 has led to immune control of HBsAg in vaccinated animals [124]. Treatment with HBsAg antibody 129G1 linking with a TLR7/8 agonist formed small molecule compound IMDQ elicits a strong and lasting anti‐HBsAg immune response after short‐term treatment in AAV/HBV mice [125]. Combination treatment with HBV‐targeted siRNA elebsiran (BRII‐835) and therapeutic vaccine BRII‐179 containing Pre‐S1, Pre‐S2, and S antigens induced HBsAg reduction, which contributed to the persistence and efficacy of HBV‐specific humoral adaptive immunity in CHB participants [126].

## 8. Conclusion

**Figure 1:**
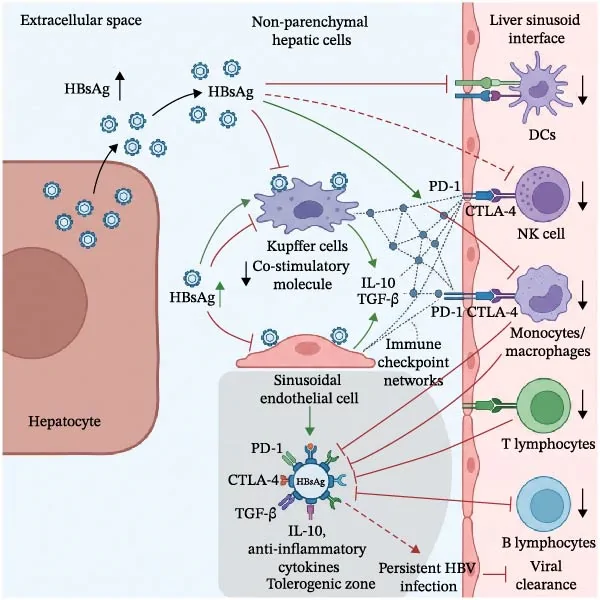
Mechanisms of immune tolerance induced by HBsAg. The regulation of HBsAg works across innate and adaptive immune responses and enhances the intrahepatic immune tolerant microenvironment.

Chronic HBV infection remains a major clinical challenge, largely due to a complex network of mechanisms that induce and maintain profound immune tolerance via HBV antigens, especially HBsAg. All these complex interactions, from innate immune system suppression to T‐ and B‐cell exhaustion and dysfunction, as well as the establishment of an immunosuppressive microenvironment in the liver, contribute to viral persistence and the paucity of a definitive cure. Therefore, re‐establishing immune competency in CHB is challenging. Strategies such as immune checkpoint blockade, therapeutic vaccines, and engineered cell therapy, which all target specific aspects of this perturbed landscape, have proved partially successful. Combination regimens of these methods show synergistic effects and thus represent a more realistic prospect of a functional cure. They are summarized in Figure 1.

## Funding

No funding was received for this manuscript.

## Conflicts of Interest

The author declares no conflicts of interest.

## Data Availability

The data that support the findings of this study are available upon request from the corresponding author. The data are not publicly available due to privacy or ethical restrictions.
